# Multi-center Korean cohort study based on RNA-sequencing data targeting COPD patients

**DOI:** 10.1038/s41597-024-03389-8

**Published:** 2024-06-06

**Authors:** Minseok Seo, Sinwoo Park, WooJin Kim, Ji Ye Jung, So Hyeon Bak, Edwin K. Silverman, Jinkyeong Park

**Affiliations:** 1 Department of Computer and Information Science, Korea University, Sejong, Republic of Korea; 2 Department of Internal Medicine, School of Medicine, Kangwon National University, Kangwon National University Hospital, Chuncheon, Republic of Korea; 3 Division of Pulmonology, Department of Internal Medicine, Institute of Chest Diseases, Severance Hospital, Yonsei University College of Medicine, Seoul, Republic of Korea; 4 Department of Radiology and Research Institute of Radiology, University of Ulsan College of Medicine, Asan Medical Center, Seoul, Republic of Korea; 5 Division of Pulmonary and Critical Care Medicine, Department of Medicine, Brigham and Women’s Hospital and Harvard Medical School, Boston, Massachusetts USA; 6 Department of Pulmonary, Allergy and Critical Care Medicine, Kyung Hee University College of Medicine, Kyung Hee University Hospital at Gangdong, Seoul, Republic of Korea

**Keywords:** Genetics, Respiratory tract diseases

## Abstract

In 2023, WHO ranked chronic obstructive pulmonary disease (COPD) as the third leading cause of death, with 3.23 million fatalities in 2019. The intricate nature of the disease, which is influenced by genetics, environment, and lifestyle, is evident. The effect of air pollution and changes in atmospheric substances because of global warming highlight the need for this research. These environmental shifts are associated with the emergence of various respiratory infections such as COVID-19. RNA sequencing is pivotal in airway diseases, including COPD, as it enables comprehensive transcriptome analysis, biomarker discovery, and uncovers novel pathways. It facilitates personalized medicine by tracking dynamic changes in gene expression in response to various triggers. However, the limited research on East Asian populations may overlook the unique nuances of COPD development and progression. Bridging this gap and using peripheral blood samples for systemic analysis are crucial for comprehensive and globally applicable COPD diagnosis and treatment.

## Background & Summary

In 2023, the World Health Organization (WHO) released a comprehensive report affirming that chronic obstructive pulmonary disease (COPD) has ascended to rank as the third foremost contributor to global mortality^1^, characterized by a heterogeneous lung condition manifested as chronic respiratory symptoms (dyspnea, cough, expectoration, and exacerbations) due to abnormalities of the airways (bronchitis, bronchiolitis) and/or alveoli (emphysema) that cause persistent, often progressive, airflow obstruction^2^. The report revealed that COPD accounted for a staggering tally of approximately 3.23 million fatalities in 2019^3^. This multifaceted disease arises from intricate interactions among genetic predisposition, environmental exposure, and lifestyle factors, leading to diverse clinical manifestations and variable treatment responses^4^. The current era, fraught with global infectious threats, such as coronavirus disease 2019 (COVID-19), indicates the urgency of comprehending the underlying pathophysiology of COPD, especially considering its heightened vulnerability to infections^5^.

In the landscape of COPD research, RNA sequencing (RNA-seq) from peripheral blood samples provides a critical lens through which we can view the systemic nature of the disease^6,7^. This approach aligns with our aim to understand COPD beyond its primary lung involvement, as it manifests systemic inflammation and immune responses that are detectable and quantifiable in blood^8^. By analyzing peripheral blood, our study captures these systemic changes, offering insights into COPD’s impact on overall health, which is especially relevant for East Asian populations exposed to unique environmental factors like biomass^9,10^ and post-tuberculosis sequelae^11^.

Our comprehensive RNA-seq analysis encompasses an extensive range of genetic expressions from protein-coding genes to noncoding RNAs and alternative splicing events, uncovering potential biomarkers and pathways critical for disease progression and response to therapies. Blood samples, which reflect systemic physiological responses to COPD, are less invasive and more accessible for patients, making them a valuable tool for broad epidemiological studies and personalized medicine. Through this method, we aim to address the lack of research in East Asian populations whose COPD characteristics may diverge from the patterns seen in Western cohorts due to distinct genetic and environmental influences.

Specifically, our study focuses on uncovering novel molecular signatures within blood that could indicate COPD in East Asians, facilitating early detection and monitoring of the disease. Identifying such biomarkers could lead to the development of targeted interventions and contribute to the nuanced understanding needed for personalized treatment strategies. Our findings reveal the roles of particular non-coding RNAs and gene expression changes related to COPD’s systemic effects, emphasizing the potential of RNA-seq in blood samples to inform targeted therapeutic interventions.

We are pioneering this inclusive approach to COPD research, which not only advances our understanding of the disease’s management and treatment but also fosters a framework for precision medicine. Considering the unique clinical and environmental context of East Asian populations, our work aims to ensure that COPD research reflects the diversity of patient experiences and meets the global health challenge with tailored solutions.

## Methods

A list of abbreviations can be found in Table 1.

**Table 1:** List of Abbreviations.

Abbreviation	Definition
CAT_SUM	Total score of COPD (Chronic Obstructive Pulmonary Disease) Assessment Test (CAT) scores
COPD	Chronic Obstructive Pulmonary Disease
COVID-19	coronavirus disease 2019
DEGs	Differentially expressed genes
DNB	DNA nanoball
FDR	False discovery rate
FEV1	Forced expiratory volume in 1 second
FVC	Forced Vital Capacity
lncRNAs	long non-coding RNAs
PCR	Polymerase chain reaction
PFT	Pulmonary Function Test
RIN	RNA integrity number
RNA-seq	RNA sequencing
RT	Reverse transcriptase
WBC	White blood cell
WHO	World Health Organization

### Ethics statement

This study was approved by the Institutional Review Board of Dongguk University Ilsan Hospital (DUIH 2020-04-012), Kyung Hee University Hospital at Gangdong (KHNMC 2022-03-063-008), Kangwon University Hospital (KNUH 2012-06-007), and Severance Hospital, Yonsei University College of Medicine (YUHS 4-2021-064). Prior to sample preparation, all samples were anonymized by assigning patient ID numbers. This study was performed in adherence to the Declaration of Helsinki.

### Patients and acquisition of blood samples

**Fig. 1:**
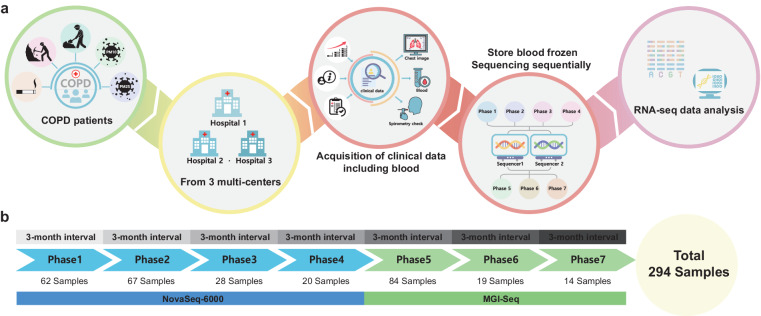
Schematic diagram to collect RNA-seq data from multi-centers and multi-platforms. **(a)** Schematic diagram of the overall study progress. **(b)** Diagram of the sample size and the experimental batches of the collected patients and the RNA-seq data.

This study was designed to compare characteristics and clinical outcomes using radiological findings and blood transcriptome in prospective COPD cohorts from three different regions: Seoul (YUHS and KHNMC), Gyeonggi province (DUIH), and Kangwon province (KNUH) in the Republic of Korea (Fig. 1a). Seoul is an urban region, Gyeonggi Province is a suburban region, and Kangwon Province is a rural area in Korea. Patients with a smoking history of more than 10 pack-years or who visited a respiratory outpatient clinic with chronic bronchitis were prospectively recruited since 2020. Patients with the following conditions were excluded from this study: (1) < 18 years of age, (2) had undergone a tracheostomy or experienced difficulty performing a pulmonary function test, (3) pregnant women, (4) previous history pulmonary resection, (5) suspected lung cancer or undergoing chemotherapy, (6) recently undergone ophthalmic surgery or been recently hospitalized for acute coronary artery disease, and (7) received radiation therapy to the thorax. Total blood RNA was collected from all participants after obtaining written informed consent to participate in the study and for their RNA data to be shared in a repository. Data on demographics, spirometry findings, imaging findings, smoking burden, respiratory symptoms and comorbidities were collected. Total RNA was collected using PAXgene™ Blood RNA tubes and extracted from all 294 samples with the Qiagen PreAnalytiX PAXgene Blood Kit (Qiagen, Valencia, CA) to ensure consistency in RNA extraction.

### Library preparation and sequencing

A total of 294 blood samples were sequentially RNA-sequenced once in the order in which blood was collected at the three hospitals (Fig. 1b). This was a prospective study in which patients visiting three hospitals were enrolled; thus, we conducted sequencing at seven different time points (Phase 1 to Phase 7). Two sequencing platforms were employed: the Illumina NovaSeq-6000 for Phases 1 to 5 and the MGISeq-2000 for Phases 6 to 7. This strategic choice aimed to broaden our data range and mitigate any bias specific to a single sequencing platform. A rigorously standardized protocol across RNA extraction and library preparation stages ensured the fidelity and comparability of our sequencing data.

The initial step of RNA extraction was meticulously conducted across all samples using the TRI-reagent-based method (QIAZOL Lysis Reagent, Qiagen, Germany), ensuring efficient and reproducible isolation of total RNA. RNA concentration and integrity were measured with the Quant-iT RiboGreen assay (Invitrogen, #R11490) and Agilent TapeStation RNA screentape (Agilent, #5067-5576), advancing only samples with an RNA integrity number (RIN) of 6.0 or higher to library construction to maintain high-quality standards for sequencing.

The library preparation for Illumina-based sequencing followed the TruSeq Stranded Total RNA protocol, leveraging the Ribo-Zero Human kit (Illumina, Inc., San Diego, CA, USA) for thorough rRNA depletion, enriching the libraries for a comprehensive array of coding and non-coding RNA species. The protocol included an initial RNA fragmentation, followed by first-strand cDNA synthesis using SuperScript II (Invitrogen, #18064014). Strand specificity was maintained in the subsequent synthesis of the second cDNA strand by incorporating dUTP instead of dTTP. Akin to this procedure, the MGISeq-2000 library preparation maintained identical steps, employing the MGIEasy RNA Directional Library Prep Set for rRNA depletion, followed by the same fragment size distribution, reverse transcriptase enzymes, and primers to preserve the strand orientation of the RNA.

Subsequent steps for both platforms—including “A” base addition, adapter ligation, and PCR enrichment—were conducted per the established Illumina protocol. Quality control for the final library involved using the same KAPA Library Quantification kits and Agilent TapeStation D1000 ScreenTape to quantify and validate the libraries, confirming uniform quality across both platforms.

Sequencing on the MGISeq-2000 employed 150-bp paired-end reads, mirroring those of the NovaSeq, to directly compare data between the platforms. Such meticulous parallelism in read length and sequencing depth between NovaSeq and MGISeq enabled us to seamlessly integrate and compare datasets, enhancing our findings’ reliability and the transcriptomic landscape’s interpretability.

### RNA-seq data processing

FastQC v.0.11.9^12^ was used to confirm the base quality of the raw reads generated by RNA-seq. Trimmomatic (v0.39) was used to remove poor bases and adapter sequences from the raw reads. The clean reads were aligned to the Grch38 human reference genome derived from the Ensembl Genome Database using Hisat2 (v2.2.1)^13^. Alignment results were recorded in sorted BAM format using Samtools view (v1.14), and mapping-related statistics were collected using Samtools stats and Hisat2 outcomes. Mapped reads were quantified using featureCounts (v2.0.1)^14^ according to the Ensembl gene annotation. All quality indicators for technical validation were organized and visualized using R software (v4.1.3). Statistical analysis was performed to identify differentially expressed genes (DEGs) using limma Voom (v3.56.2)^15^. In a statistical hypothesis test to find differentially expressed genes according to recent smoking status (Current vs Former/Never smokers), in addition to the main effect (current smoking status), seven different sequencing batches, gender, age, height, and weight, were considered as covariates. Similarly, in the analysis of differentially expressed genes between two different sequencing platforms (Nova-seq and MGI-seq), modeling was performed with the same covariate correction in addition to the two sequencing platform information as the main fixed effect. In this study, the false discovery rate (FDR) adjustment method was used to correct for multiple testing problems^16^, and a 5% significance level was considered as the cutoff.

## Data Records

Raw and preprocessed RNA-seq data were deposited in the GEO database under the accession number GSE24065615^17^. Quality control data, preprocessed data, clinical phenotypes, and R codes can be found on figshare^18^.

## Technical Validation

In our investigation of the COPD patient cohort, we performed a rigorous quality control analysis on RNA-seq data sourced from blood samples of 294 individuals. These patients were selected based on their proximity to cement factories and coal mines in South Korea, smoking history, or exposure to other environmental pollutants. A total of 294 patients were enrolled in the study, with 83.3% reporting a smoking history averaging 26.7 pack-years. These details, along with other demographic information such as age and sex distribution, which mirrors established COPD cohorts, are presented in Table 2. The mean age was 68.9 years with a majority being male (79.6%). This demographic breakdown supports the comparability of our study with existing literature^19,20^. Pulmonary function tests indicated that 54.4% of patients experienced airflow limitations, with 16.6% displaying moderate-to-severe airflow limitation, reflected by an average FEV1 of 2.14 liters. A summary of these clinical findings is provided in the Table 2, which includes the COPD status and spirometry results.

**Table 2:** Baseline Characteristics of enrolled patients.

	Overall	Never smoker	Ever smoker
**n**	294	49	245
**Age**	68.89 ± 13.59	72.18 ± 16.04	68.24 ± 12.98
**Gender = M (%)**	234 (79.6)	5 (10.2)	229 (93.5)
**Height**	163.61 ± 9.07	151.88 ± 7.58	165.95 ± 7.37
**Weight**	65.55 ± 11.33	59.04 ± 10.38	66.85 ± 11.08
**Current smoke**	100 (34.0)	0 (0.0)	100 (40.8)
**Smoke_amount**	26.74 ± 21.89	0.00 ± 0.00	32.53 ± 19.86
**Airflow limitation**	160 (54.4)	9 (18.4)	151 (61.6)
**Spirometry Grade (%)**			
** 0**	99 (33.7)	30 (61.2)	69 (28.2)
** 1**	111 (37.8)	9 (18.4)	102 (41.6)
** 2**	48 (16.3)	0 (0.0)	48 (19.6)
** 3**	1 (0.3)	0 (0.0)	1 (0.4)
** U**	35 (11.9)	10 (20.4)	25 (10.2)
**Enrolled PFT**			
**FEV1/FVC**	65.12 ± 14.91	76.87 ± 8.06	62.89 ± 14.87
**FEV1**	2.14 ± 0.82	1.65 ± 0.44	2.23 ± 0.85
**FVC**	3.32 (1.01)	2.14 (0.46)	3.55 (0.92)
**WBC**	6969.45 ± 1907.57	6493.88 ± 1495.93	7064.96 ± 1968.70
**Neutrophil(%)**	57.06 ± 8.99	55.56 ± 9.31	57.37 ± 8.91
**Lymphocyte(%)**	31.28 ± 8.38	34.20 ± 8.62	30.70 ± 8.23
**Monocyte(%)**	7.70 ± 2.07	7.30 ± 1.79	7.78 ± 2.12
**Eosinophil(%)**	2.93 ± 2.27	2.28 ± 1.44	3.06 ± 2.38
**Basophil(%)**	0.67 ± 0.32	0.65 ± 0.32	0.67 ± 0.33
**Eosinophil_count**	199.58 ± 171.94	141.35 ± 81.29	211.32 ± 182.79
**Monocyte_count**	525.56 ± 169.97	469.74 ± 145.64	536.81 ± 172.55
**Lymphocyte_count**	2144.88 ± 749.37	2187.54 ± 671.17	2136.27 ± 765.16
**Neutrophil_count**	4027.06 ± 1510.97	3653.24 ± 1245.42	4102.75 ± 1550.52
**Hemoglobin**	14.07 ± 6.03	12.12 ± 1.43	14.46 ± 6.51
**Hematocrit(%)**	41.29 (5.36)	36.28 (3.65)	42.28 (5.09)
**CAT_SUM**	15.50 ± 8.67	19.02 ± 9.19	14.81 ± 8.42

Each participant’s sample, subjected to sequencing, met strict quality standards: RIN greater than 6 and a minimum concentration of 25 μg/ul, ensuring the integrity of our RNA data. The sequencing, detailed in Fig. 1, was conducted on two platforms, NovaSeq and MGI-seq, with each sample being sequenced once to eliminate the possibility of batch effects and ensure consistent data. The sequencing was methodically phased into seven parts, corresponding with the sample collection order, to maintain RNA integrity and reduce degradation risks.

**Fig. 2:**
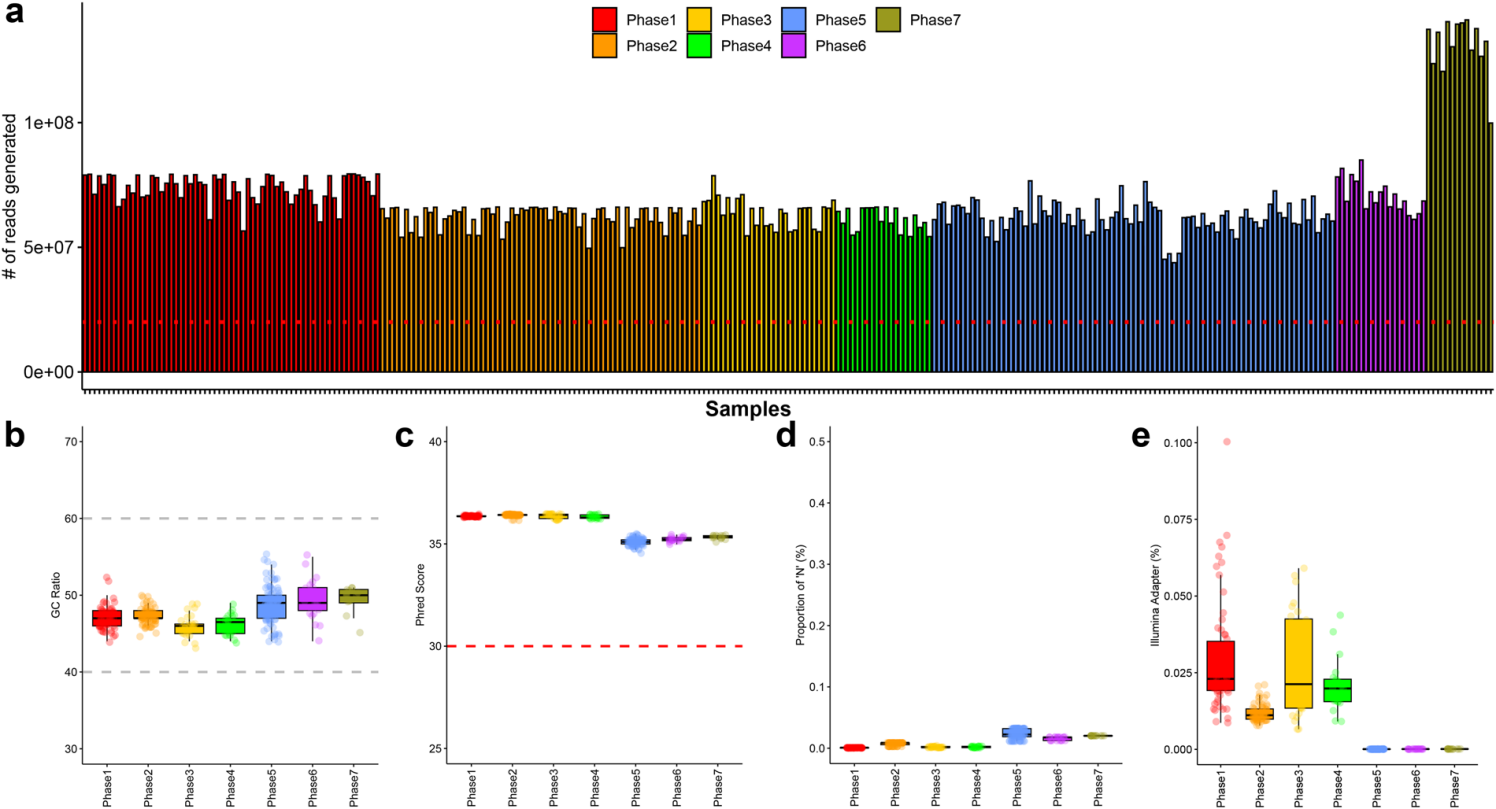
Quality control of raw reads generated through RNA-sequencing. In all plots, colors represent different sequencing batches. **(a)** Number of raw generated reads across all samples. **(b)** G/C ratio per each sequencing batch. **(d)** Phred scores of raw reads generated from each sequencing batch. **(d)** Proportion of unsequenced bases among total raw reads generated from each sequencing batch. **(e)** Illumina’s universal adapter discovery rate per each sequencing batch.

The average number of raw reads generated across the entire RNA-seq dataset was 68,610,798 reads per sample (range: 43,812,379–141,160,930 reads) (Fig. 2a). Considering that the recommended number of reads in RNA-seq research is over 20 million^21,22^, we confirmed that a sufficient number of raw reads were generated across all samples. For technical verification of the generated raw reads, we checked their basic characteristics using fastQC software. A review of the generated reads’ GC ratio confirmed no special sequencing GC bias at 47.724% across batches, which aligns with the expected genomic range and shows no particular sequencing bias (Fig. 2b). It is widely known that the percentage of GC in generated reads should ideally be close to 50% to minimize sequencing bias. Using the Phred score, an index that can intuitively indicate sequencing quality, we confirmed that all samples had a base call accuracy of ≥99.9%, which was 35.883, reflecting the high quality of our sequencing data (Fig. 2c). We found that “N” contained an average of 0.01%, and a slightly higher ratio was observed in the MGI-seq platform (Fig. 2d).

**Fig. 3:**
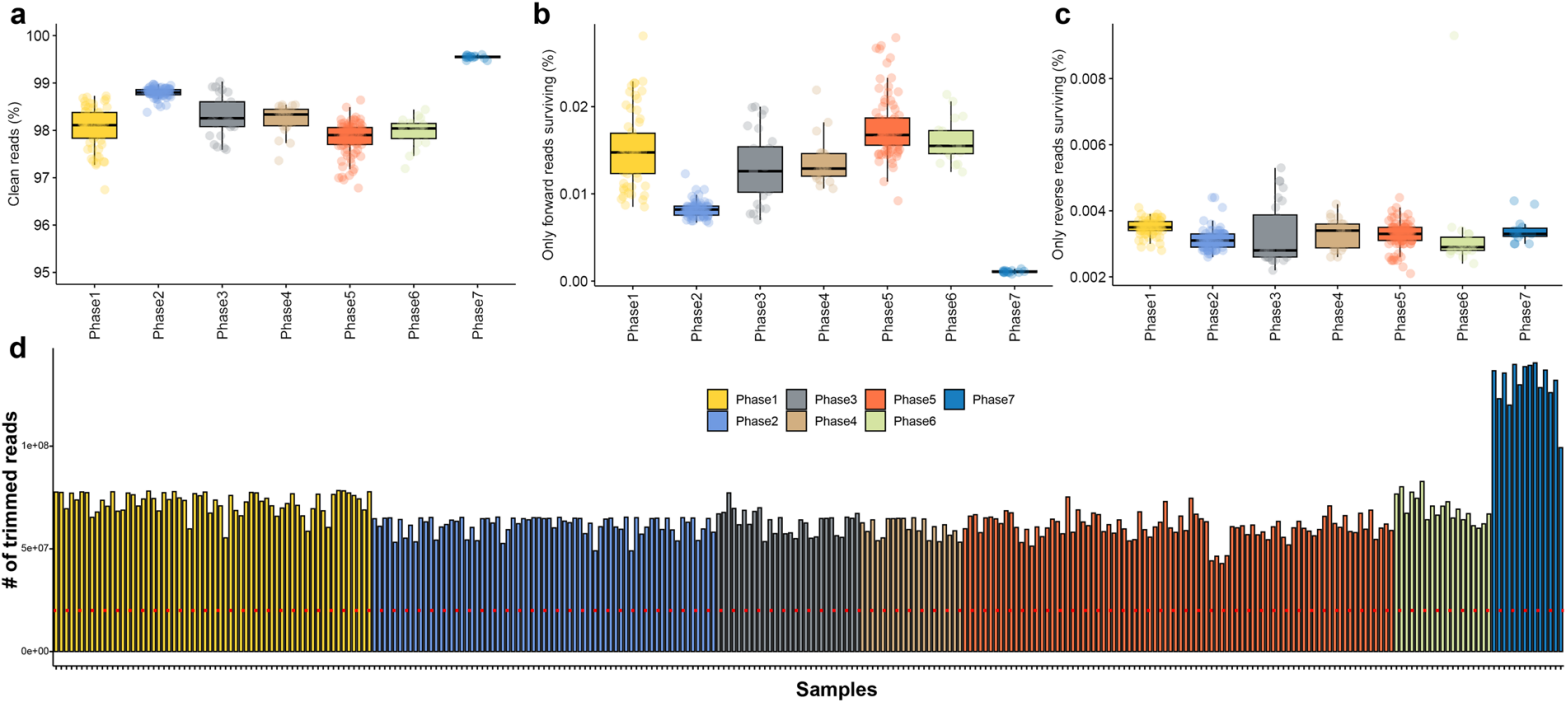
Statistics of cleaned reads after quality control process through Trimmomatic. In all plots, colors represent different sequencing batches. **(a)** Proportion of reads that passed the filtering process among all raw reads. **(b)** Ratio of only forward reads among paired-end reads passing the filtering process. **(c)** Ratio of only reverse reads among paired-end reads passing the filtering process. **(d)** Number of clean reads that passed the filtering process across the entire samples.

When examined for inclusion of universal adapters from Illumina based on the FastQC tool, adapter sequences were detected only in samples included in phases 1 to 4 generated using the Illumina sequencing platform. As expected, no Illumina universal adapter seuqence was detected in samples from stages 5 to 7 sequenced via MGI-seq, indicating that all samples were sequenced as planned without mislabeling issues (Fig. 2e). Particularly noteworthy was the high quality of reads in phase 2, as highlighted in Fig. 3a and b. While the reasons for the elevated quality in this phase are multifaceted—ranging from variations in sample collection to operational shifts in the sequencing facilities—these did not impact the overall integrity of our research findings. Our rigorous QC standards across all phases ensured data reliability and robustness, and the minor variations observed did not influence the conclusions of our research.

After completing the technical validation of the raw reads generated through RNA-seq, we proceeded to remove adapter sequences that might be attached to the ends of the reads and eliminate reads of poor quality using the Trimmomatic software. On average, 98.259% of the raw reads successfully passed through this filtering process (Fig. 3a). Among the paired-end reads, only forward reads survived at a rate of 0.013% (Fig. 3b), and only reverse reads survived at a rate of 0.003% (Fig. 3c) across all the samples. Due to their very low numbers, these surviving reads were excluded from the subsequent alignment step. We primarily utilized the paired-end information, which is relatively reliable. After confirming the number of cleaned reads that remained following the removal of low-quality bases and adapter sequences, it was technically confirmed that the average number of reads was 67,452,913, exceeding the expected number of 20 million reads (ranging from 42,843,793 to 149,526,153 reads) (Fig. 3d).

**Fig. 4:**
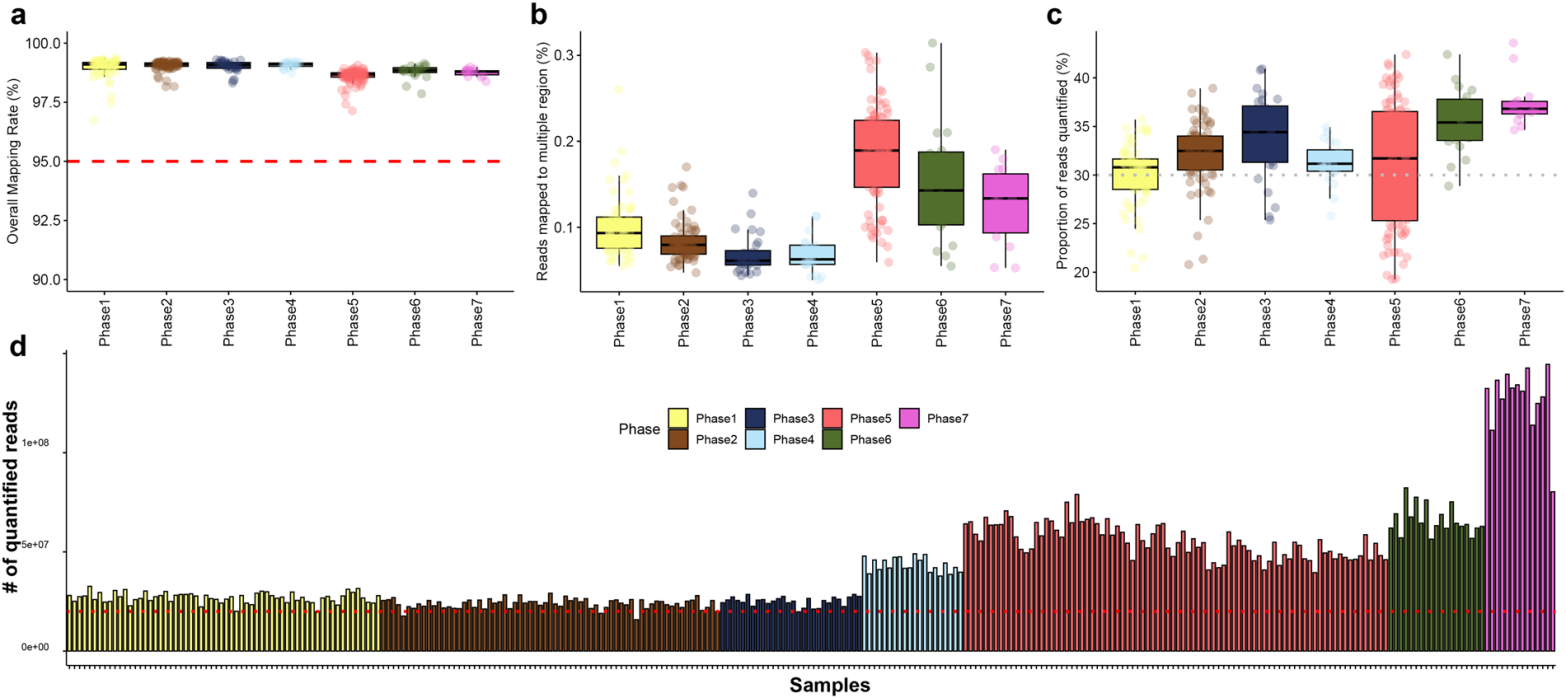
Mapping and quantification quality Based on the GRCH38 reference genome and gene annotation. In all plots, colors represent different sequencing batches. **(a)** Proportion of clean reads successfully mapped to the Grch38 human reference genome. **(b)** Percentage of mapped reads to multiple regions (two or more) on the reference genome. **(c)** Proportion of reads quantified as specific transcripts based on Ensembl gene annotation. **(d)** Number of reads successfully quantified across entire samples.

As a result of aligning these quality-verified cleaned reads to the human reference genome, an average mapping rate of 98.867% was observed, indicating excellent sequencing quality (Fig. 4a). While most sequencing quality indicators did not significantly differ between the two sequencing platforms, a notable discrepancy was observed in the multiple alignment ratio during the process of mapping reads to the reference genome (Fig. 4b). On average, the ratio of multiple mapped reads for reads generated through the Nova-seq platform was 0.085, whereas for reads generated via MGI-seq, it was 0.171. This is a relative characteristic that may occur during library preparation in the entire process of securing RNA-seq samples. After the technical validation of mapping quality, we proceeded to the final step, gene annotation-based quantification. As we targeted the entire transcriptome and adopted a conservative approach that did not consider multiple mapped reads during the quantification process, we expected an assignment rate of 30% at the gene level during quantification based on the current quality control outcome of benchmark study^23^. Upon quantification based on Ensembl human gene annotation, it was confirmed that an average of 31.981% of mapped reads were successfully quantified across all samples (Fig. 4c). In total, an average of 42,611,086 mapped reads were quantified as expression of specific human genes emphasizing the high-quality quantification process that was carried out (Fig. 4d).

**Fig. 5:**
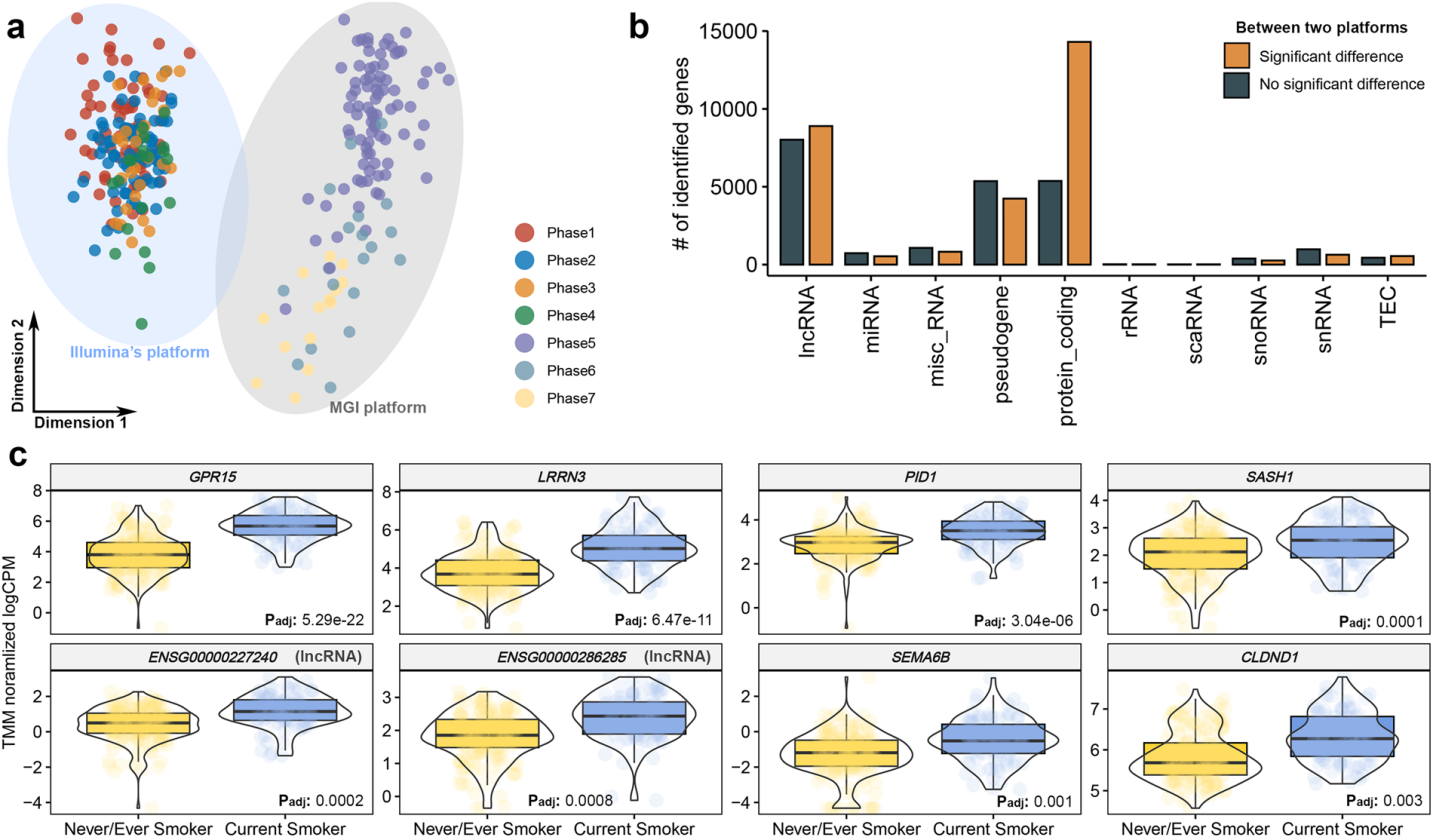
Identifying differentially expressed genes between two different sequencing platforms or smoking status. **(a)** The result of dimensionally reducing the expression levels of all quantified genes using the multi-dimensional scaling method. **(b)** Investigation of types of differentially expressed genes in two sequencing platforms. The RNA type used information described in Ensembl biomart. DEGs identified between the two sequencing platforms were determined at a 5% significance level after FDR adjustment. **(c)** Top 8 genes whose expression levels significantly change depending on current smoking status.

Following the execution of dimensionality reduction analysis to assess the spread of 294 samples, for which expression were measured across a total of 57,791 genes, significant disparities in expression were discerned between the two sequencing platforms (Fig. 5a). It was substantiated that 32,600 genes, which comprises more than half of the total genes (56.41%), exhibited notable variations in expression between the two sequencing platforms at a significance level of 5%, after FDR adjustment (Supplementary Data S1). In our exploration of whether genes demonstrating differences in expression depending on the sequencing platform could be characterized according to detailed gene types outlined in the Ensembl gene annotation, we identified an enrichment of protein-coding genes (Fig. 5b and Supplementary Data S2). When designing this study, we hypothesized that distinctions might arise across different sequencing platforms due to various experimental factors, including differences in the library preparation process, biotechnological characteristics of sequencing, and other related variables. Although previous studies have yet to be able to consider experimental bias factors stemming from disparities in sequencing technologies, mainly owing to the high costs associated with next-generation sequencing technology, this study attempted to consider that. As a result, we suggest the possibility that the relative expression of transcripts could be measured differently depending on the sequencing technology and/or library preparation method used. We expect that this observation will be revealed in the near future through research in the field of biotechnology that confirms expression levels by only varying the conditions of specific experimental stages.

It is well known that the key risk factor for COPD, which is the primary subject of this study, is smoking history. Although large-scale RNA-seq studies examining the association between COPD and smoking status in Asian populations are limited, there are many outcomes from large-scale studies reporting Western populations, such as COPDGene^24^. Within our cohort, we included a diverse range of smoking statuses to examine the effects of smoking on gene expression comprehensively. Our comparative analysis encompassed 294 samples from both smokers and non-smokers, providing a broad representation of the population. Additionally, we paid particular attention to current smokers, with 100 samples from this subgroup undergoing sequencing. This allowed us to draw more nuanced comparisons and identify genes differentially expressed with current smoking status. Current smokers were compared to both former smokers and never smokers. This approach enabled us to examine the differential gene expression associated with current smoking status in comparison to both groups, offering a comprehensive understanding of the effects of smoking on gene expression.

In this study, we sequenced 294 samples, utilizing two different sequencing platforms to enhance the robustness of our data. 177 samples were sequenced using the Illumina NovaSeq-6000 platform, and 117 samples were sequenced using the MGISeq-2000 platform. This dual-platform strategy was instrumental in broadening our analytical scope and ensuring that platform-specific biases did not limit our findings. Our dataset identified 20 genes that exhibited differential expression according to the current smoking status, with statistical significance at the 5% level after adjusting for the FDR (Supplementary Data S3). As expected, the top five genes discovered in this study were markers validated in previous large-scale transcriptome studies based on smoking status. For example, the three top genes identified in this study (Fig. 5c), *GPR15* (P_adj_:5.29e-22), *LRRN3* (P_adj_:6.47e-11), and *PID1* (P_adj_:3.04e-06), were also identified as representative biomarkers that were differentially expressed depending on smoking status in the ECLIPSE study, an international representative cohort for COPD^25^. Moreover, among the identified markers, *CLDND1* (P_adj_:0.003), *LRRN3*, and *SASH1* (P_adj_:0.0001) were identified as biomarkers related to smoking status using microarray and qRT-PCR techniques in a previous study^26^. This provides direct evidence that the generated data are technically valid.

We further verified whether findings such as ENSG00000286285 lncRNA, which was not reported to be associated with current smoking status, were East Asian-specific based on large-scale RNA-seq data from an independent Western cohort. To clarify this observation, we performed the same analysis using RNA-seq data from COPDGene, the largest representative cohort studying COPD in Western populations. As a result, five representative genes, *GPR15* (P_adj_:5.93e-38), *LRRN3* (P_adj_:4.19e-36), *PID1* (P_adj_:3.98e-19), *CLDND1* (P_adj_:3.88e-21), and *SASH1* (P_adj_:1.68e-17), which are differentially expressed according to current smoking status revealed in this study, were most significantly found in the Western population as well^27^. Furthermore, it was technically verified that *ENSG00000286285* lncRNA showed no significant difference according to the current smoking status in the study for the Western population.

In the quest to unravel the complexity of COPD and its diverse manifestations, our study has identified several novel lncRNAs that may serve as potential biomarkers in the East Asian population. To validate these lncRNAs, we are extending our research through planned collaborations with established Korean cohorts, including Korean Obstructive Lung Disease (KOLD)^28^ and Korean COPD Subgroup Study (KOCOSS)^29^, and with the international COPDGene project. Such collaborations will allow us to validate our findings across varied population subsets. These efforts aim to confirm the potential of these novel lncRNAs as universal or population-specific biomarkers, offering an unprecedented depth of understanding of COPD in various racial and environmental settings. We anticipate that this collaborative work will solidify the foundation of our study, contributing to the global initiative of tailoring COPD management and therapeutic interventions to individual patient profiles.

### Supplementary information

3rd_revision_scientific_dataR

Supplementary Data S1

Supplementary Data S2

## Data Availability

The R code used to perform differential expression analysis is available in figshare^18^.
